# Supplementary material for: HIF-1α Expression Increases Preoperative Concurrent Chemoradiotherapy Resistance in Hyperglycemic Rectal Cancer
Source: Cancers (Basel). 2022 Aug 22;14(16):4053. doi: 10.3390/cancers14164053 (PMC9406860; doi:10.3390/cancers14164053)
Supplement: Supplementary file 1 [file cancers-14-04053-s001.zip › cancers-1816583-supplementary.pdf]

Supplemental Figure S1.

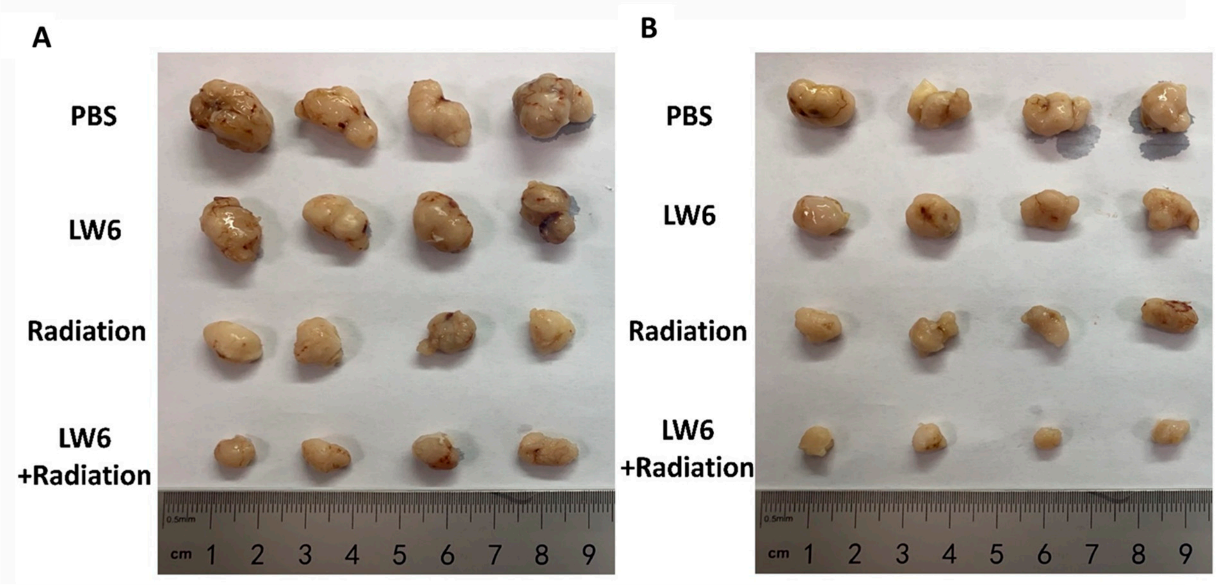

Supplemental Figure S1. The tumor picture of the effect of combined LW6 on the radiation of hyperglycemic rectal cancer. Hyperglycemic ectopic rectal cancer was treated with LW6, radiation, or combined LW6 with radiation. At day 30, the mice were sacrificed and the tumors (A, HCT116; B, SW480) were collected to observe.
